# Aortic regurgitation in athletes: the challenges of echocardiographic interpretation

**DOI:** 10.1186/s44156-023-00033-w

**Published:** 2023-12-13

**Authors:** Nikhil Chatrath, Jamie O’Driscoll, Sanjay Sharma, Michael Papadakis

**Affiliations:** Cardiovascular Clinical Academic Group, St George’s, University of London, Cranmer Terrace, London, SW17 0RE UK

**Keywords:** Aortic regurgitation, Bicuspid aortic valve, Athlete, Exercise

## Abstract

**Background:**

Bicuspid aortic valve (BAV) is the most common congenital cardiac defect and prone to premature degeneration causing aortic regurgitation (AR). The assessment of AR in athletic individuals poses several challenges as the pathological left ventricle (LV) remodelling caused by AR may overlap with the physiological remodelling of intense exercise. The purpose of this study is to highlight these challenges, review the existing literature and discuss how to tackle these conundrums. As a real-world example, we compare the resting transthoracic echocardiographic (TTE) findings in a cohort of individuals with BAV and AR, sub-grouped into “highly active” or “lightly active”.

**Methods:**

Adult male subjects with an index TTE performed at a tertiary referral centre between 2019 and 2022 were included if the TTE confirmed a BAV and at least moderate AR. Further strict inclusion criteria were applied and parameters of valve disease severity was made in accordance with existing guidelines. Subjects completed a physical activity questionnaire over the telephone, and were classified into either group 1: “highly active” or group 2: “lightly active” based on their answers. Demographics and TTE parameters were compared between the two groups.

**Results:**

30 male subjects (mean age 44 ± 13 years) with BAV-AR were included – 17 were highly active, and 13 lightly active. There was no significant difference in age (group 1, 45 ± 12.7 years vs. group 2, 42 ± 17 years; p = 0.49), height (p = 0.45), weight (p = 0.268) or severity of AR, when quantitative assessment was possible. Group 1 had a significantly higher stroke volume (131 ± 17mls vs. 102 ± 13 mls; p = 0.027), larger LV volumes, diastolic dimensions and significantly larger bi-atrial and right ventricular size. This LV dilatation in the context of AR and athleticism poses a diagnostic and management conundrum. Despite this, none of these 17 highly active individuals demonstrated any of the traditional criteria used to consider surgery.

**Conclusion:**

There is significant overlap between the physiological adaptations to exercise and those caused by AR. Multi-modality imaging and stress testing can aid clinicians in diagnostic and management decisions in exercising individuals when there is discordance between AR severity and symptoms.

## Background

Bicuspid aortic valve (BAV) is the most common cardiac defect, with an estimated prevalence of 1–2% in the general population [1]. It is prone to premature degeneration and as such, BAV-related aortic regurgitation (AR) is one of the most common valvular disorders encountered in young individuals.

The assessment of AR in athletic individuals poses several challenges as the pathological left ventricular (LV) remodelling caused by AR may overlap with the physiological remodelling of intense exercise. LV dilatation and low-normal or mildly impaired LV systolic function can be both features of severe AR requiring surgical intervention and athletic adaptation in an endurance athlete [2]. This is particularly relevant in BAV as quantifying AR severity poses additional challenges due to the eccentricity of the regurgitant jet. Finally, the physician needs to consider that athletic individuals are likely to have reserves way above sedentary counterparts in terms of symptoms and therefore careful assessment of exercise performance and fitness levels with both subjective and objective measures is necessary.

The aim of this study is to describe the transthoracic echocardiogram (TTE) findings in individuals with significant BAV-related AR (BAV-AR) and make comparisons between athletic versus sedentary individuals. By doing so, we aim to to highlight the challenges, review the existing literature and promote discussion on how to overcome these challenges.

## Methods

Adult subjects (> 16 years old) with an index TTE performed at a tertiary referral centre between January 2019 and January 2022 were included if the TTE confirmed a BAV and at least moderate AR. The specific inclusion and exclusion criteria are shown in Table 1. TTE measurement technique, parameters of valve disease severity and measurement indexing was made in accordance with current British Society of Echocardiography (BSE) guidelines [3]. Given the intrinsic differences in cardiac chamber geometry between sexes, and the lack of specifically highly-active females with BAV-AR seen at our institution, only male subjects were included in this study to allow direct comparison, thereby negating the confounder of sex.

**Table 1:** List of inclusion and exclusion criteria

Inclusion criteria	Exclusion criteria
Age > 16 or <65 years	Age ≤ 16 or ≥ 65 years
≥Moderate AR defined by > 1 of: • Regurgitant fraction > 30% • Regurgitant volume > 30mls • Vena contracta width ≥ 0.3 cm • Pressure half time ≤ 500ms • Descending aorta end-diastolic velocity ≥ 20 cm/s • Dense jet width CW • Large jet width colour flow • Diastolic flow reversal in descending aorta (intermediate or prominent holodiastolic) • Large flow convergence colour flow When ≤ 1 of the above criteria not met, severity assessed by ≥ 2 experienced sonographers/echocardiologists	>Mild aortic stenosis >Mild mitral regurgitation >Mild tricuspid regurgitation
Sinus rhythm	Atrial fibrillation
Completed exercise questionnaire	Known concomitant cardiomyopathy (including dilated or hypertrophic cardiomyopathy)
	Ischaemic heart disease with previous MI or PCI
	History of BAV endocarditis
	Previous cardiac surgery
	Previous cardiac surgery
	Poor imaging quality

*CW* continuous wave, *MI* myocardial infarction, *PCI* percutaneous coronary intervention

Subjects were consented to complete a telephone questionnaire pertaining to their current and previous exercise levels. Subsequent quantification of each activity into metabolic equivalents (METs) was performed, as outlined by the Compendium of Physical Activities [4]. Subjects with a history of ≥ 3 h/week of vigorous exercise (≥ 6 METs) for ≥ 2 years were classified as highly active, similar to definitions in previous studies of athletic individuals with hypertrophic cardiomyopathy [5]. Those with < 1 h vigorous exercise per week were classified as “lightly active”. Demographics and TTE parameters were compared between the two groups.

Statistical analyses were performed using SPSS 2021 edition (IBM, New York). Results are mean ± standard deviation (SD) for continuous variables and comparisons between groups made using Student *t*-test. Formal ethical approval was obtained from the Health Research Authority (HRA) in order to complete the exercise questionnaire (REC reference number 22/SC/0358).

## Results

273 individuals with BAV had their index TTE performed at our institution between January 2019 and January 2022. The majority were male (72% (199/273). From the male cohort, 30 subjects met the inclusion criteria, mean age 44 ± 13 years (range 19 to 62). According to the aforementioned classification, 13 subjects were lightly active (group 1) and 17 subjects highly active (group 2). In group 1, the majority had moderate AR (8/13), 4/13 moderate-severe AR and 1/13 severe AR. In group 2, 10/17 had moderate AR and 7/17 had moderate-severe AR.

In the highly active cohort, the vast majority of individuals performed activities with a predominantly high dynamic, low static component [6]. The predominant exercise included running (6), football (5), cycling (3), tennis (1), boxing (1) and mixed martial arts (1). They performed on average 1432 Met-min/week of vigorous exercise. The lightly active group performed on average 32 met-min/week of vigorous exercise with 9/13 not undertaking any vigorous exercise at all.

The differences between the two groups are shown in Table 2. There was no significant difference in terms of age (group 1, 45 ± 12.7 years vs. group 2, 42 ± 17 years; p = 0.49), height (p = 0.45), weight (p = 0.268) or severity of AR, when quantitative assessment was possible (AR pressure half time p = 0.89 and vena contracta p = 0.85).

Highly active individuals had a significantly lower resting heart rate (58 ± 7 bpm vs. 71 ± 13 bpm; p = 0.004), higher stroke volume (131 ± 17mls vs. 102 ± 13 mls; p = 0.027) and significantly larger left ventricular volumes and diastolic dimensions (Table 2). Highly active individuals also had evidence of enlargement of the other cardiac chambers with significantly larger bi-atrial and right ventricular sizes.

**Table 2:** Echocardiographic parameters in the overall cohort, and sub-divided into group 1 (lightly active) and group 2 (highly active)

	Overall	Group 1 (lightly active)	Group 2 (highly active)	p-value
Subjects (n)	30	13	17	
*Demographics*				
Age (years)	44 (± 13)	45 (± 13)	42 (± 14)	0.49
Height (cm)	180 (± 7)	179 (± 8)	181 (± 6)	0.41
Weight (kg)	83 (± 11)	85 (± 10)	80 (± 11)	0.27
Heart rate (bpm)	64 (± 12)	71 (± 14)	59 (± 7)	**< 0.01**
*Left ventricle*				
LVEDD (mm)	56 (± 5)	53 (± 5)	59 (± 3)	**< 0.001**
LVEDDi (mm/m²)	25.9 (± 5.9)	24.0 (± 5.0)	26.8 (± 4.9)	**0.12**
LVESD (mm)	37 (± 5)	35 (± 4)	38 (± 6)	0.13
LVESDi (mm/m²)	16.5 (± 4.9)	15.1 (± 5.1)	17.2 (± 4.9)	0.26
LVEDVi (ml/m²)	83 (± 23)	66 (± 14)	96 (± 19)	**< 0.001**
LVESVi (ml/m²)	34 (± 14)	27 (± 11)	39 (± 13)	**0.01**
Ejection fraction (%)	61 (± 6)	61 (± 5)	61 (± 7)	0.80
GLS (%)	-18.3 (± 2.0)	-18.6 (± 1.9)	-17.8 (± 2.2)	0.30
E/E’ average	7.1 (± 2.6)	7.3 (± 1.0)	7.0 (± 3.2)	0.79
Stroke volume (mls)	118 (± 36)	102 (± 33)	131 (± 34)	**0.03**
Cardiac output (l/min)	7.48 (± 2.33)	7.23 (± 2.89)	7.67 (± 1.87)	0.61
*Aortic valve*				
AV peak velocity (m/s)	2.21 (± 0.69)	1.95 (± 0.41)	2.42 (± 0.81)	0.07
AV mean velocity (m/s)	1.69 (± 0.79)	1.37 (± 0.28)	1.95 (± 0.96)	0.05
AV VTI (cm)	48.07 (± 17.89)	39.9 (± 9.03)	54.3 (± 20.58)	**0.03**
AR pressure half-time (ms)	486 (± 134)	481 (± 148)	489 (± 131)	0.89
Vena Contracta width (cm)	0.52 (± 0.15)	0.51 (± 0.19)	0.53 (± 0.11)	0.85
LVOT peak gradient (mmHg)	1.10 (± 0.22)	1.03 (± 0.15)	1.15 (± 0.25)	0.15
*Aorta*				
Sinus of valsalva index (mm/m)	20.9 (± 2.8)	21.0 (± 3.3)	20.8 (± 2.5)	0.83
Sinotubular junction index (mm/m)	18.8 (± 2.9)	18.4 (± 3.5)	19.2 (± 2.5)	0.45
Ascending aorta index (mm/m)	21.1 (± 3.6)	21.4 (± 4.5)	20.9 (± 2.9)	0.71
*Other chambers*				
Left atrium volume index (ml/m²)	37.1 (± 22.7)	24.8 (± 6.5)	46.6 (± 26.2)	**0.02**
RA area index (cm/ m²)	8.9 (± 3.3)	7.4 (± 1.8)	10.4 (± 3.3)	**0.02**
RV basal diameter (mm)	40 (± 7)	36 (± 8)	43 (± 5)	**0.007**
RV mid diameter (mm)	31 (± 6)	30 (± 7)	32 (± 5)	**0.01**

Values in bold indicate statistical significance

Parameters written as mean ± standard deviation (SD). *LVEDD* left ventricular end-diastolic dimension, *LVEDDi* LVEDD indexed to BSA, *LVESD* left ventricular end-systolic dimension, *LVESDi* LVESD indexed to BSA, *LVEDVi* left ventricular end-diastolic volume index, *LVESVi* left ventricular end-systolic volume index, *GLS* global longitudinal strain, *AV* aortic valve, *AR* aortic regurgitation, *LVOT* left ventricular outflow tract

## Discussion

This study highlights the challenges in using TTE to determine the haemodynamic impact of AR on the LV in highly active individuals. Similar to healthy athletes, in our cohort, the highly active individuals with AR demonstrated larger cardiac chambers, which may lead to overestimation of the impact of the AR, cause undue concern and potentially lead to earlier intervention [7]. Our study, offers some reassurance as none of the 17 highly active individuals demonstrated any of the traditional criteria used to consider surgery such as LVESD > 50 mm or > 25 mm/m^2^ or resting LV EF ≤ 50%.

However, it is these adaptations which make it challenging to unpick from the pathological adaptations of severe AR and will be discussed below.

 What are the challenges?

1. *Bradycardia:* Bradycardia causes lengthening of diastole, thereby prolonging the LV diastolic filling time. Resultantly, as AR is a diastolic process, one might expect larger LVEDVs in those with bradycardia, as seen in our cohort.
2. *LV dilatation*: It is well recognised that LV dilatation occurs in response to the augmented loading conditions induced by repetitive bouts of particularly dynamic exercise [2]. LV dilatation should promote consideration of surgical intervention when accepted cut-offs are met (LVESD > 50 mm or 25 mm/m² [8]). In our cohort, the LVEDD was significantly larger in highly active individuals (p < 0.05) whereas the LVESD was not (p = 0.13). However both the LVEDVi and LVESVi were significantly larger (both p < 0.05). This suggests that even when LVESD cut-offs for severity are not met, the LVESV volumes may be significantly increased, which could suggest underestimation of the AR severity and its long-term impact on the individual. Recent evidence suggests that mortality in subjects with asymptomatic moderate/severe AR is significantly increased for LVESDi > 20 mm/m² [9]. Further studies are required assessing for similar outcomes using volumes (LVESDV) instead of dimensions (LVESD), before volume cut-offs makes their way into guideline recommendations.
3. *Stroke volumes*: The average stroke volume (SV) of 118mls in the overall AR cohort is higher than the 70-100mls expected in the normal adult heart. This is a reflection of the increased loading conditions of AR. However, in our cohort, the highly active individuals had a significantly higher SV than their less active counterparts. Though this could be due to the severity of AR not actually being similar in the two groups, it could also be due to the additive effect of high intensity exercise on the LV loading conditions.
4. *Ejection fraction*: Athletic individuals often have a more efficient ventricle, thereby requiring a lower EF to generate the same cardiac output. In our cohort, there was no difference in EF between the two groups, with an LVEF of 61% in the highly active group, despite their larger LV volumes. This suggests that the AR is possibly causing more of a haemodynamic effect on the LV, with increased contractility to compensate for the larger volume. It is only in the later stages of chronic AR that the LV systolic function begins to impair, and is an indication for surgical intervention [8]. Once the LV function deteriorates, symptoms can begin rapidly [10]. One could thus infer that that a low-normal EF in an athlete with > moderate AR should require prompt evaluation, and not should not be attributed to their athleticism.
5. *AR eccentricity*: BAV-AR tends to be highly eccentric in nature, sometimes with multiple jets and thus conventional quantitative markers of AR severity are often not applicable to this cohort. One relies on qualitative assessment, for which there may be significant intra-observer variability. As a result, one may look for other markers of severity or alternative imaging modalities.

What are the solutions?

1. *Multi-modality imaging*
  AR may be under-estimated by TTE alone. Particularly for the highly eccentric jets of BAV-related AR, there is an incremental value of using 2D and 3D transoesophageal echocardiography (TOE) for further evaluation [11]. Cardiac magnetic resonance (CMR) can quantify transaortic flows with accuracy and reproducibility [12]. Based on CMR findings, a previous study reclassified AR severity as non-severe in 34% of subjects graded as having severe AR by TTE [13]. CMR has the added benefit of accurate LV volume quantification and tissue characterisation. It has identified interstitial fibrosis in up to 10% of individuals with AR, irrespective of the AR severity [14].
  Many individuals with chronic AR may develop aortic root or ascending aorta dilatation, particularly in those with BAV where aortopathy is common [15]. Though TTE and TOE can provide multi-planar imaging of the aorta, CMR and CT provide high resolution imaging of the aorta without limitation by acoustic window and are thus the recommended imaging modalities.
2. *Stress testing* 
  Exercise testing, can unmask symptoms, reveal exercise-induced arrythmias and provide an objective assessment of physical fitness and is thus a crucial tool used to provide an individual with exercise recommendations in the presence of significant AR [16, 17]. Cardiopulomary exercise testing (CPET) would provide this information and parameters can be assessed and compared on follow-up visits.
  Using exercise stress echocardiography (ESE) to quantify AR severity however is not recommended, as the shorter diastolic time caused by the ensuing tachycardia, invariably leads to an improvement in the AR severity [18]. This has been demonstrated in multiple studies, which explains why even significant AR can be well tolerated and not negatively impact sporting performance, as long as the LV function is preserved [19].
  When performed, ESE should therefore focus on specific LV parameters. An absence of contractile reserve (CR) (defined as an > 5% increase in LVEF on exercise) has been shown to be a better predictor of LV decompensation after surgery than resting indices of LV function [20]. More recently, in asymptomatic individuals with severe AR and preserved LV function, an absence of CR was shown to be independently associated with deterioration of symptoms or LV systolic function [21]. In this study, one third of patients with larger LV dimensions demonstrated adequate CR, whereas one third of patients with smaller LV dimensions did not have CR. This second group did not qualify for surgery based on current recommendations of LV size and suggests that ESE may be able to further stratify individuals for aortic valve replacement [21, 22].
3. *Follow-up*
  In some cases the dilemma of moderate or severe AR and its resultant haemodynamic impact on the LV may remain despite comprehensive evaluation. In such individuals, follow-up is crucial as progressive LV dilatation or dysfunction or even subtle reduction of fitness levels detected by CPET, may be suggestive of an earlier need for surgery, even if established criteria are not met [23]. In such cases comparison between the same imaging modality and objective assessment of fitness levels with cardiopulmonary exercise testing should be considered. In athletic individuals a period of detraining may be considered to differentiate the influence of exercise form that of AR on the LV. It is important, however, to consider that detraining is problematic particularly in competitive athletes who commonly do not adhere due to the effects of deconditioning. Moreover, it may lead to false conclusion as studies in elite athletes with increased LV wall thickness and cavity size suggest that up to 20% may continue to exhibit significant cavity dilatation after detraining [24].

## Study limitations

This is a single centre study in a tertiary referral centre for sports cardiology, which is prone to referral bias and may account for the greater number of highly active compared with lightly active individuals. It is limited by a small sample size, with only males included so one can not extrapolate these findings to wider populations. Patients were grouped according to their answers to an exercise questionnaire which has inherent issues with recall bias. Moroever, longitudinal follow-up is not provided to assess longer term outcomes and test the validity of our practice and suggestions. However, the primary aim was to describe findings on an individuals’ index resting TTE.

## Conclusion

There is significant overlap between the physiological adaptations to exercise and those caused by AR. TTE remains the cornerstone of AR assessment in athletic individuals but it has significant limitations, particularly in BAV-AR which is one of the most common valvular disorders encountered by sports cardiologists. Multi-modality imaging and stress testing can aid clinicians in diagnostic and management decisions in exercising individuals when there is discordance between AR severity and symptoms.

## Data Availability

The datasets used and/or analysed during the current study are available from the corresponding author on reasonable request.
